# Gait Analysis Using Walking-Generated Acceleration Obtained from Two Sensors Attached to the Lower Legs

**DOI:** 10.3390/s25144527

**Published:** 2025-07-21

**Authors:** Ayuko Saito, Natsuki Sai, Kazutoshi Kurotaki, Akira Komatsu, Shinichiro Morichi, Satoru Kizawa

**Affiliations:** 1 Department of Mechanical Science and Engineering, Kogakuin University, 2665-1 Nakanomachi, Hachioji 192-0015, Tokyo, Japan; 2 Graduate School of Engineering, Kogakuin University, 2665-1 Nakanomachi, Hachioji 192-0015, Tokyo, Japan; 3 Department of General Engineering, National Institute of Technology (KOSEN), Sendai College, 48 Nodayama, Medeshima-Shiote, Natori-shi 981-1239, Miyagi, Japan; akomatsu@sendai-nct.ac.jp; 4 Department of Pediatrics and Adolescent Medicine, Tokyo Medical University, 6-7-1 Nishishinjuku, Shinjuku-ku 160-0023, Tokyo, Japan; smorichi@tokyo-med.ac.jp; 5 Department of Mechanical Engineering and Robotics, National Institute of Technology (KOSEN), Akita College, 1-1 Iijima-Bunkyo-cho, Akita 011-8511, Akita, Japan; kizawa@akita-nct.ac.jp

**Keywords:** centrifugal acceleration, gait analysis, gravitational acceleration, lower leg, sensor fusion, tangential acceleration, translational acceleration

## Abstract

Gait evaluation approaches using small, lightweight inertial sensors have recently been developed, offering improvements in terms of both portability and usability. However, accelerometer outputs include both the acceleration that is generated by human motion and gravitational acceleration, which changes along with the posture of the body part to which the sensor is attached. This study presents a gait analysis method that uses the gravitational, centrifugal, tangential, and translational accelerations obtained from sensors attached to the lower legs. In this method, each sensor pose is sequentially estimated using sensor fusion to combine data obtained from a three-axis gyroscope, a three-axis accelerometer, and a three-axis magnetometer. The estimated sensor pose is then used to calculate the gravitational acceleration that is included in each axis of the sensor coordinate system. The centrifugal and tangential accelerations are determined from the gyroscope output. The translational acceleration is then obtained by subtracting the centrifugal, tangential, and gravitational accelerations from the accelerometer output. As a result, the acceleration components contained in the outputs of the accelerometers attached to the lower legs are provided. As only the acceleration components caused by walking motion are captured, thus reflecting their characteristics, it is expected that the developed method can be used for gait evaluation.

## 1. Introduction

Gait evaluation approaches using small, lightweight sensors have recently been developed, offering improvements in terms of both portability and usability [1,2,3]. For example, [4] demonstrated the high validity and test–retest reliability of a novel gait assessment system that employs convolutional neural networks to extract three-dimensional skeletal joint data from monocular frontal-view videos of walking individuals. Similarly, [5] highlighted the potential of smartphone-based gait analysis to reduce the workload and complexity of applications in fields such as healthcare, sports, rehabilitation, and fitness. The effectiveness of motion analysis using wearable devices for evaluating the physical condition of patients has been proven in rehabilitation and clinical settings. Accurate measurement of body movements—particularly through sensors such as accelerometers—is essential for capturing the characteristics of human motion and ensuring reliable gait assessment. Several studies have analyzed acceleration patterns during walking using a minimal number of inertial sensors. For instance, [6] evaluated gait improvements in patients with mobility impairments by proposing a gait quality index (GQI), calculated from the power spectrum of vertical trunk acceleration. The GQI was defined as the ratio of total spectral power up to 10 Hz to the power associated with gait-specific components, serving as an indicator of gait quality. In another study, [7] investigated longitudinal changes in trunk acceleration, gait speed, and paretic leg motion in post-stroke hemiparetic patients. They examined inter-variable relationships over time and whether initial trunk acceleration metrics could predict gait speed after two months. Their results indicated that the root mean square (RMS) of trunk acceleration correlated with gait speed, stride length, and the trailing limb angle. In research using an Explainable Artificial Intelligence approach [8], DL models—including a convolutional neural network (CNN) and a gated recurrent unit (GRU)—were trained using 1-stride and 8-stride accelerations, respectively, to classify adult and older adult groups. In particular, the abovementioned gait assessment approaches using acceleration capture the characteristics of gait patterns using the time course of acceleration. Accelerometers can detect unusual temporal patterns that are not visually identifiable and can be used to identify abnormal gaits and extract the features of individual gaits. However, accelerometer outputs include both the acceleration that is generated by human motion and gravitational acceleration, which changes with the posture of the body part to which the sensor is attached.

To analyze the acceleration generated during walking, several studies have attempted to decompose the accelerometer output into components. For example, [9] investigated whether differences exist in upper-body acceleration patterns between young and elderly individuals during natural-speed walking. In their study, gravitational acceleration was corrected by mathematically removing it based on the tilt angle of the accelerometer when the subject was standing still. However, this correction method is limited, as it cannot adequately compensate for gravitational acceleration when the sensor-mounted body segment undergoes significant tilting during walking. To address this limitation, [10] proposed a mathematical algorithm for removing the gravitational component from accelerometer data. In their approach, the tilt angle of the body segment to which the accelerometer is attached is measured using a gyroscope, and the gravitational acceleration is sequentially subtracted from the sensor output in accordance with changes in segment orientation. This enables the extraction of dynamic acceleration due to body movement—i.e., excluding gravitational effects—for subsequent use in gait evaluation. However, this correction method relies on gyroscopes, which are susceptible to drift over long-term measurements due to bias instability. Alternatively, [11] proposed an optimization-based method to isolate gravitational acceleration from the accelerometer signal. In this approach, gait evaluation was performed by analyzing the periodic pattern of the gravitational component, which was estimated using characteristic frequencies derived through frequency analysis of acceleration data during steady walking. While this method can successfully extract the gravitational acceleration, it remains limited in terms of its ability to evaluate the dynamic motion of body segments, as the observed gravitational acceleration primarily reflects posture changes rather than motion-specific characteristics of the sensor-mounted body part(s).

The acceleration that is generated by human motion includes centrifugal, tangential, and translational accelerations, as discussed in previous studies [12,13,14]. Centrifugal and tangential accelerations describe the movement of joints that are adjacent to the part of the body to which the sensor is attached, whereas translational acceleration describes the movement of joints more proximal to the sensor location. This means that, by attaching a sensor to a distal part of the body and decomposing the acceleration into centrifugal, tangential, and translational accelerations, it is possible to obtain the movement characteristics of the body part to which the sensor is attached, as well as other parts of the body, using only a single sensor. However, while the centrifugal and tangential accelerations can be calculated from the angular velocity measured using a gyro sensor, calculation of the translational acceleration requires acceleration information from all sensors attached to the proximal part. To calculate the translational acceleration in a simpler way, it is necessary to subtract the centrifugal, tangential, and gravitational acceleration components from the output of the distally attached accelerometer.

Therefore, nine-axis motion sensors were attached to the lower legs of healthy subjects to measure their normal gait in this study. The sensor pose was sequentially estimated by combining the outputs of a three-axis gyroscope, a three-axis accelerometer, and a three-axis magnetometer. In this arrangement, the gyroscopes measure the centrifugal and tangential accelerations generated when walking, while the gravitational acceleration can be obtained from the estimated sensor pose. Then, the translational acceleration generated when walking can be obtained by removing the gravitational, centrifugal, and tangential components from the measured acceleration. This study examines the acceleration components contained in the output of an accelerometer attached to the lower leg and identifies the characteristics of acceleration caused only by walking movements. Although derivation of the acceleration attributable solely to walking motion is based on approaches from the existing literature, the present method aims to facilitate portable gait evaluation through the isolation of motion-specific acceleration components.

## 2. Gait Measurement

### 2.1. Experimental Conditions

**Figure 1:**
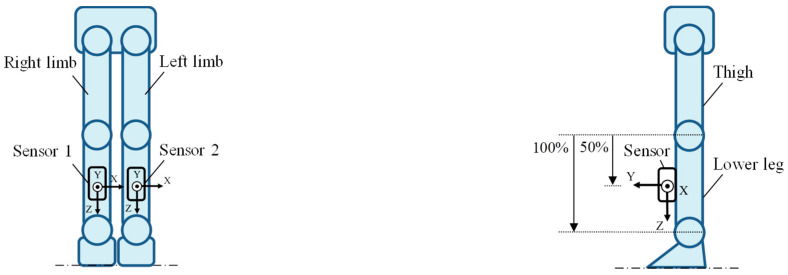
Sensor positions and sensor coordinate system.

**Figure 2:**
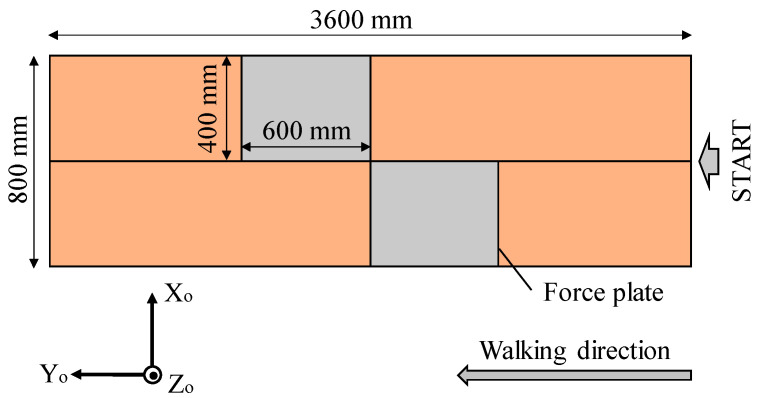
Walking path and reference coordinate system.

Five healthy adult males (height 1.72 ± 0.02 m, weight 64.2 ± 6.9 kg) participated in the experiment. The inclusion criteria required the participants to be in good health and to possess height and body composition (i.e., BMI) representative of the average adult Japanese male population. After receiving an explanation of the purpose and requirements of the study, the participants gave their written informed consent to participate. Study approval was obtained from the Research Ethics Board of Kogakuin University (Approval number 2022-B-44). The sensor positions and the sensor coordinate system are presented in Figure 1. The walking path in the laboratory and the reference coordinate system are presented in Figure 2. The two nine-axis motion sensors (SS-WS1792, Sports Sensing Co., LTD, Fukuoka, Japan) were attached at the midpoints of the front surfaces of the lower left and right legs of the participants [11,15,16]. The sensor position was the midpoint of the lower leg. The length of the lower leg was defined according to previous research [17]. There are two reasons for the choice of the lower leg as the location to which the sensor was attached. The first is that, by attaching the sensor to the lower leg distal to the knee joint, the flexion and extension movements of the knee joint can be inferred from the centrifugal and tangential acceleration components of the acceleration sensor output. The second is that the acceleration caused by the rotational movement of the more proximal joints and center of gravity movement is captured as translational acceleration of the lower leg, meaning that the output of the acceleration sensor attached to the lower leg can be used to infer the movement of more proximal parts. In the experiment, we measured the “normal gait”; that is, the natural walking gait of the participant. The participants maintained an upright position for 5 s after the start of the measurement and then began walking.

They were instructed to walk using a natural stride in time with a metronome (90 bpm). The measurement was continued for 5 s after the participant reached the end of the walking path. The sampling frequency of the nine-axis motion sensors, 3D motion analysis system (Bonita10, Vicon Co., Ltd., Oxford, UK), and force plates was 100 Hz.

### 2.2. Joint Angle Results

**Figure 3:**
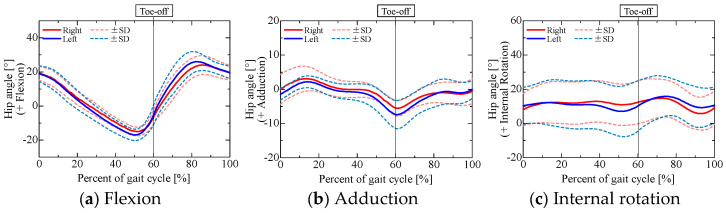
Hip angles obtained from 3D motion analysis system.

**Figure 4:**
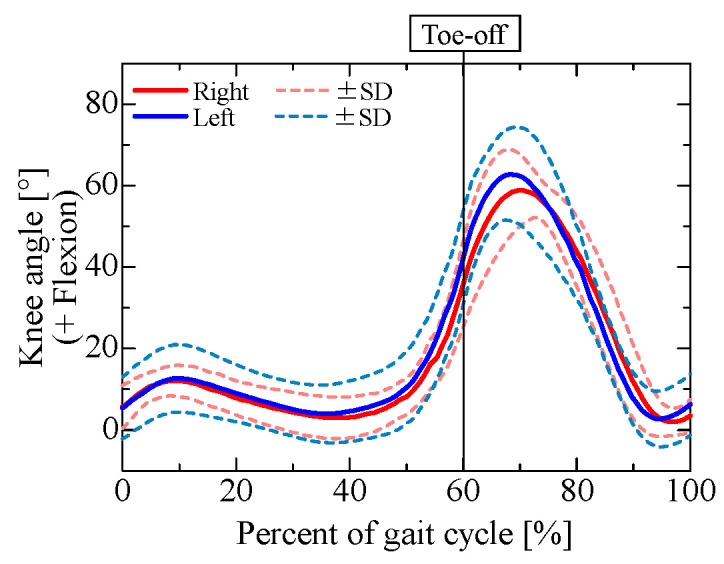
Knee flexion angle obtained from 3D motion analysis system.

**Figure 5:**
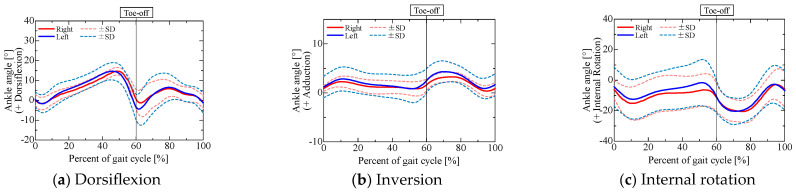
Ankle angles obtained from 3D motion analysis system.

After capturing gait data from the participants, we examined the hip, knee, and ankle joint angles obtained from a 3D motion analysis system, as shown in Figure 3, Figure 4 and Figure 5.

In Figure 3, Figure 4 and Figure 5, the red and blue curves indicate the results for the right and left limbs, respectively, and the solid and dashed curves indicate the average and standard deviation of the joint angles, respectively, over all trials and participants (3 trials for each of the 5 participants, for a total of 15 trials). The horizontal axis shows the normalized time for one gait cycle, with the times at which the foot touched the walking path indicated by 0% and 100%. The left and right toe-off times shown in each graph indicate the average toe-off time over all trials, and the measurement data were linearly interpolated by dividing them into the stance and swing phases based on the average toe-off time.

The results show the same tendency for joint angle variation during normal gait as found in earlier studies [18,19,20]. Because there was little variation between trials and between subjects, the results indicate that the same walking movements were performed in all trials.

## 3. Gravitational, Centrifugal, Tangential, and Translational Acceleration

The centrifugal, tangential, and translational accelerations of the sensors attached to the lower leg were derived using the gyroscope, accelerometer, and magnetometer included in the nine-axis motion sensor. This section first presents the method for deriving each of these acceleration components. The gravitational component is derived from estimated sensor orientations using established sensor fusion methods [21,22,23,24,25], which allow for the integration of data from a gyroscope, an accelerometer, and a magnetometer. Centrifugal and tangential accelerations are calculated from gyroscope measurements. Translational acceleration is then isolated by subtracting the gravitational, centrifugal, and tangential components from the raw accelerometer signals.

### 3.1. Centrifugal and Tangential Accelerations

The centrifugal acceleration Aceni and tangential acceleration Atani in the sensor coordinate system are, respectively, given by:

(1) Aceni=ωi×ωi×ri=−ωy2−ωz2rx+ωxωyry+ωxωzrzωxωyrx+−ωz2−ωx2ry+ωyωzrzωxωzrx+ωyωzry+−ωx2−ωy2rz,

(2) Atani=ω˙i×ri=−ωz˙ry+ωy˙rzωz˙rx−ωx˙rz−ωy˙rx+ωx˙ry,

where ωi is the gyroscope output, ω˙i is its time derivative, ri is the position vector for the sensor (with the center of the knee joint defined as the origin in the sensor coordinate system), and × indicates vector multiplication. The gyroscope output is differentiated in the Laplace domain as follows:

(3) $$D=\frac{s}{1+ns},$$

where s is the Laplace domain variable and n (=0.01) is the time constant.

For the participants in the experiment, the position vector components for the sensor (with the center of the knee joint defined as the origin in the sensor coordinate system) were $r_{x}$ = 0 m, $r_{y}$ = −0.05 m, and $r_{z}$ = −0.215 ~ −0.190 m.

### 3.2. Gravitational and Translational Accelerations

The accelerometer output Ai is expressed as the sum of the translational acceleration Atrai, the centrifugal acceleration Aceni, the tangential acceleration Atani, the Coriolis acceleration Acoli, and the gravitational acceleration gi in the sensor coordinate system as follows:

(4) Ai=Atrai+Aceni+Atani+Acoli+gi.

When an accelerometer is attached to the lower leg, the position vector from the center of the knee joint to the accelerometer is almost constant. Thus, the Coriolis acceleration (Acoli=2ωi×r˙i) can be considered to be 0. The translational acceleration can be obtained by removing the centrifugal, tangential, and gravitational accelerations from the accelerometer output. Of these, the centrifugal and tangential accelerations can be calculated from Equations (1) and (2), respectively, and the gravitational acceleration can be obtained from the estimated sensor pose. The sensor pose was estimated for a combined nine-axis sensor [21,22] consisting of a three-axis gyroscope, a three-axis accelerometer, and a three-axis magnetometer.

The 3D posture of the sensor is represented using the roll *ϕ*, pitch *θ*, and yaw *ψ* angles about the *x*-, *y*-, and *z*-axes, respectively, in a right-handed reference coordinate system with a vertical *z*-axis. Counterclockwise rotation is defined as positive. As the initial roll and pitch angles cannot be obtained from the gyroscope output, the initial values were calculated using the gravitational acceleration obtained from the accelerometer at rest [23,24]. The initial roll and pitch angles ϕAi and θAi in the sensor coordinate system are, respectively, given by:

(5) ϕAi=arctanAyiAzi (−π<ϕAi<π),

(6) θAi=arctan−AxiAyi2+Azi2 (−π<θAi<π),

where Axi, Ayi, and Azi denote the *x*-, *y*-, and *z*-axis components of the accelerometer output in the sensor coordinate system, respectively.

The initial yaw angle ψAi can be calculated using the magnetometer output. Correcting the yaw angle [25] requires the roll ϕAi, pitch θAi, and magnetometer output, as follows:

(7) mxc,imyc,imzc,i=cosθAisinϕAisinθAicosϕAisinθAi0cosϕAi−sinϕAi−sinθAisinϕAicosθAicosϕAicosθAimximyimzi

where mxi, myi, and mzi, respectively, denote the *x*-, *y*-, and *z*-axis components of the magnetometer output, and mxc,i, myc,i, and mzc,i are the respective *x*-, *y*-, and *z*-axis components of the corrected magnetic field in the sensor coordinates. The initial yaw angle can then be obtained from the corrected magnetic field as:

(8) ψmi=arctan−myc,imxc,i (−π<ψmi<π).

The differential equations for the roll, pitch, and yaw angles in the sensor coordinate system are given by:

(9) ψ˙iθ˙iϕ˙i=0sinϕisecθicosϕisecθi0cosϕi−sinϕi1sinϕϕitanθicosϕitanθiωxiωyiωzi,

where ϕ˙i, θ˙i, and ψ˙i, respectively, represent the time derivatives of the roll, pitch, and yaw angles, and ωxi, ωyi, and ωzi, respectively, represent the *x*-, *y*-, and *z*-axis components of the gyroscope output in the sensor coordinate system. Then, the roll, pitch, and yaw angles can be calculated by substituting Equation (9) into the following equation:

(10) ψiθiϕit+1=∫tt+1ψ˙iθ˙iϕ˙idt+ψiθiϕit,

where the angle vectors on the left- and right-hand sides consist of the roll, pitch, and yaw angles at time *t* + 1 and time *t*, respectively.

The Kalman filter is a widely used algorithm for estimating the true state of a system in the presence of measurement noise. In pose estimation, it enhances accuracy by effectively filtering noise from sensor data such as accelerometer and gyroscope measurements. This approach is particularly advantageous when fusing data from multiple sensor modalities. Accordingly, this study employs the Kalman filter to perform attitude estimation.

A nonlinear discrete-time system for sensor pose estimation was constructed. The nonlinear state equation was developed using Equation (10), and the nonlinear observation equation was developed using the yaw angle calculated from the magnetometer and accelerometer outputs. These two equations are, respectively, given by:

(11) xt+1i=Fi(xti,ωti)+wti,

(12) yti=Hi(xti)+vti,

where

xti=ψiθiϕit,

Fi(xti,ωti)=ψti+sinϕtisecθtiωy,ti⋅Ts+cosϕtisecθtiωz,ti⋅Tsθti+cosϕtiωy,ti⋅Ts−sinϕtiωz,ti⋅Tsϕti+ωx,ti⋅Ts+sinϕtitanθtiωy,ti⋅Ts+cosϕtitanθtiωz,ti⋅Ts,

yti=ψmiAxiAyiAzit,

Hi(xti)=ψtiR0i,tTgo,

R0i,t=cosψti−sinψti0sinψticosψti0001⋅cosθti0sinθti010−sinθti0cosθti⋅1000cosϕti−sinϕti0sinϕticosϕti,

go=009.8.

In the above equations, ϕi, θi, and ψi, respectively, denote the roll, pitch, and yaw angles in the sensor coordinate system estimated using the extended Kalman filter; ωx,ti, ωy,ti, and ωz,ti, respectively, denote the *x*-, *y*-, and *z*-axis components of the gyroscope outputs; *Ts* is the sampling time; ψmi is the yaw angle calculated using Equation (8); Axi, Ayi, and Azi, respectively, denote the *x*-, *y*-, and *z*-axis components of the accelerometer output; R0i is the rotation matrix from the reference coordinate system to the sensor coordinate system; go is the gravitational acceleration in the reference coordinate system; and wti and vti represent white noise.

The extended Kalman filter is a variant of the Kalman filter that is specifically designed for estimating the states of nonlinear systems. It facilitates the application of the Kalman filtering framework by approximating nonlinear functions via local linearization. In this process, differentiation is essential, as it yields the Jacobian matrices that are required for linearizing the state transition and observation models. To solve the extended Kalman filter, the partial derivatives of fi(xti,ωti) and hi(xti) are obtained as:

(13) fi(xti,ωti)=∂Fi(xti,ωti)∂xti,

(14) hi(xti)=∂Hi(xti)∂xti.

Then, the prediction and filtering steps are calculated using the nonlinear discrete-time system given in Equations (11) and (12). The prediction step is described by:

(15) xt+1−i=Fi(xti,ωti),

(16) Pt+1−i=ftiPtiftiT+Qti,

and the filtering step by:

(17) Vt+1i=yt+1i−Hi(xt+1−i),

(18) Bt+1i=ht+1iPt+1−iht+1iT+Rti,

(19) Kt+1i=Pt+1−iht+1iTht+1iPt+1−iht+1iT+Rti−1,

(20) xt+1i=xt+1−i+Kt+1iyt+1i−Hixt+1−i,

(21) Pt+1i=I−Kt+1iht+1iPt+1−i,

where Pi is the error covariance matrix, Vi denotes the prediction error matrix, Bi denotes the prediction error variance matrix, Ki is the Kalman gain, Qi is the covariance matrix for the process noise wti in the nonlinear state equation, and Ri is the covariance matrix for the observation noise vti in the nonlinear observation equation. In this study, the noise covariance matrices Qi and Ri were determined from the sensor output and adjusted for the error caused by sensor noise and the changes in dynamics over time [22]. In [22], the process and observation noise covariance matrices in the extended Kalman filter were determined based on the state–space model dynamics and the sensor noise. The postural change appears in the gyroscope output due to the rotational motion of the joints produced during human movement. Consequently, the process noise covariance matrix was determined, based on the gyroscope output, as follows:

(22) Qti=Ωω,ti000Ωω,ti000Ωω,ti,

where

Ωω,ti=aω2x,ti+ω2y,ti+ω2z,ti+b,

In these expressions, ωx,ti, ωy,ti, and ωz,ti, respectively, stand for the gyroscope output in the *x-*, *y-*, and *z*-axes, while *a* and *b* are adjusting parameters. In this study, *a* and *b* were determined through maximizing the log-likelihood (LLi) shown in Equation (25):

(23) LLi=−N2ln(2π)−12∑j=1NlnBji+Vji2Bji

where *N* stands for the number of time-series data and *j* represents the time-series index. In addition, Bji expresses the prediction error variance, and Vji is the prediction error.

The observation noise covariance matrices must be set at a high value when the sensor noise increases [21]. Therefore, the observation noise covariance matrix was determined based on the accelerometer and magnetometer output, as these two sensor outputs were used as observation values [26]. The observation noise covariance matrix is presented below:

(24) Rti=Ωm,ti0000Ωa,ti000000Ωa,ti00Ωa,ti,

where

Ωm,ti=c(mc,ix,t2+mc,iy,t2+mc,iz,t2−m¯)+d,

Ωa,ti=e(Aix,t2+Aiy,t2+(Aiz,t−g)2)+f,

In the equations above, mc,ix,t, mc,iy,t, and mc,iz,t, respectively, denote the corrected magnetic field data for the *x*-, *y*-, and *z*-axes; $\bar{m}$ represents the average value of the magnetometer output over the entire measurement time; ***^i^A_x,t_***, ***^i^A_y,t_***, and ***^i^A_z,t_***, respectively, express the accelerometer outputs for the *x-*, *y-*, and *z*-axes; and *c*, *d*, *e*, and *f* are adjusting parameters. In particular, *c*, *d*, *e*, and *f* were also determined by maximizing the log-likelihood (LL) shown in Equation (23).

The gravitational acceleration included in the accelerometer output in the sensor coordinate system was calculated using the sensor pose obtained via sensor fusion:

(25) git=R0i,tTgo,

where git denotes the gravitational acceleration in the sensor coordinate system.

The translational acceleration can then be obtained by removing the centrifugal acceleration Aceni, tangential acceleration Atani, and gravitational acceleration gi from the accelerometer sensor output in the sensor coordinate system using Equation (4), as follows:

(26) Atrai=Ai−Aceni+Atani+gi.

Here, the Coriolis acceleration (Acoli=2ωi×r˙i) is assumed to be 0 as the position vector ri from the knee joint center to the accelerometer is almost constant. Thus, the translational acceleration is derived by removing the centrifugal, tangential, and gravitational accelerations from the accelerometer output.

**Figure 6:**
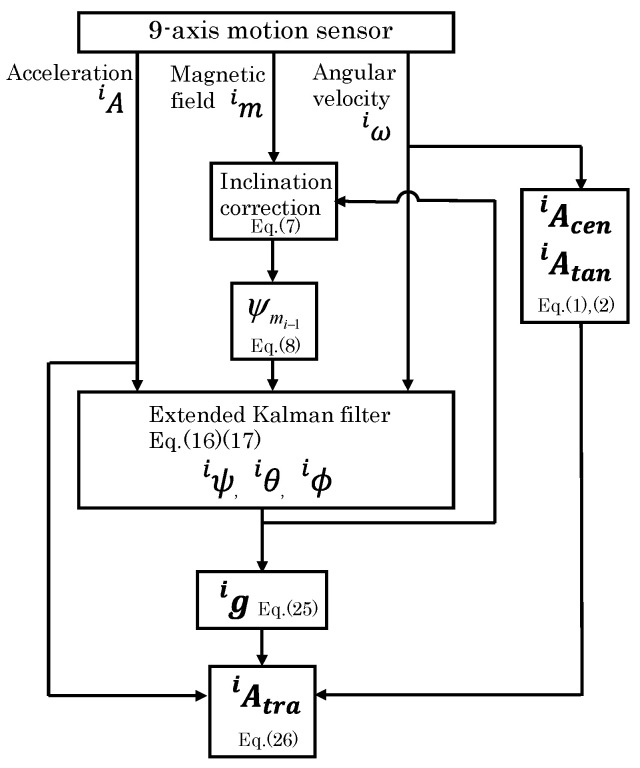
Workflow for calculating each acceleration.

A workflow diagram for calculating each acceleration is shown in Figure 6. Scilab (Scilab6.1.0, Dassault Systèmes Co., Ltd., Paris, France) was used to implement the algorithms for their calculation.

### 3.3. Sensor Pose Estimation Results

**Figure 7:**
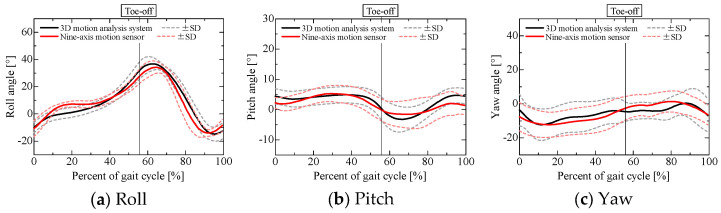
Sensor pose of right lower leg estimated using nine-axis motion sensor.

**Figure 8:**
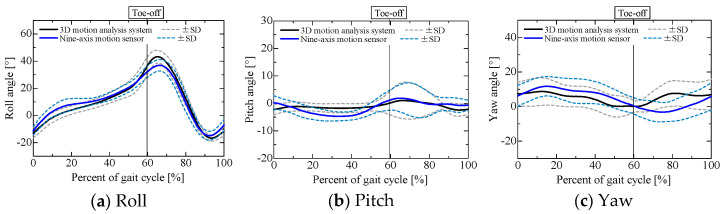
Sensor pose of left lower leg, estimated using nine-axis motion sensor.

Before evaluating the centrifugal, tangential, and translational accelerations, we examined the accuracy of the estimated sensor pose obtained through sensor fusion when using a nine-axis motion sensor, as shown in Figure 7 and Figure 8, in comparison with the pose obtained using a 3D motion analysis system. The angles obtained from the 3D motion analysis system represent the posture angles of the lower leg segments. Table 1 lists the root mean square errors for the results obtained from the nine-axis motion sensor and the 3D motion analysis system. The roll, pitch, and yaw angles are the angles about the *x*-, *y*-, and *z*-axes of the reference coordinate system shown in Figure 2, respectively, with counterclockwise rotation taken as positive. Note that the angle for each axis when each participant was standing still was set to 0°.

Although the pitch angle during the stance phase and the yaw angle during the swing phase for sensor 2 attached to the left lower leg were slightly less accurate than the other estimated results, the errors in the roll and yaw angles were about 5°, while the error in the pitch angle was about 2°. This estimation accuracy is comparable to that reported in previous research [22]. A comparison of the estimated sensor pose and the lower limb joint angles obtained from the optical motion capture system confirmed that the sensor pose was estimated with reasonable accuracy.

The abovementioned estimation accuracy is also better than those reported in comparative analyses against modern deep-learning-based fusion methods. In pose estimation using recurrent neural networks (RNNs) [27], the accuracy rate of the test data was approximately 84% after about 36 min of training based on a long short-term memory recurrent neural network (LSTM-RNN). Pose estimation using a transformer for time-series filtering [28], utilizing link length derivatives, mean interpolation, and median filtering to detect and interpolate jitter, switching, and false positives, yielded errors in the order of tens of degrees when compared to joint angle measurements using optical motion capture. Thus, this model cannot yet achieve a usable level of accuracy in situations where precise joint angles are required. A single-stage pose estimation algorithm named yolov8-sp [29] improves the original yolov8 architecture through the incorporation of multi-dimensional feature fusion and an attention mechanism to automatically determine feature importance. Using this method, joint angles were detected in various sports scenarios, and the overall joint angle detection accuracy was 89%.

In the next section, we therefore describe the characteristics of each gait using the centrifugal and tangential accelerations and the translational acceleration calculated using the sensor pose.

### 3.4. Acceleration Results

**Figure 9:**
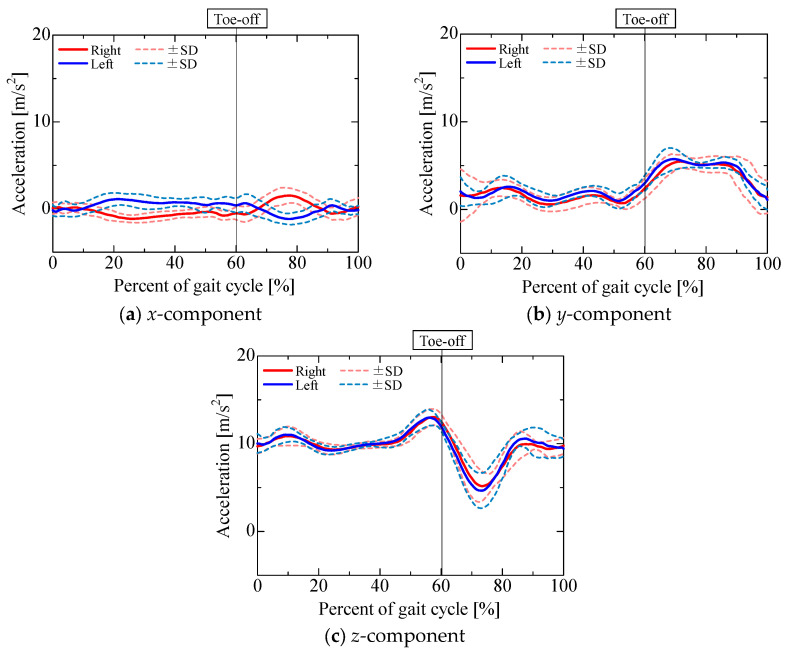
Accelerometer output.

**Figure 10:**
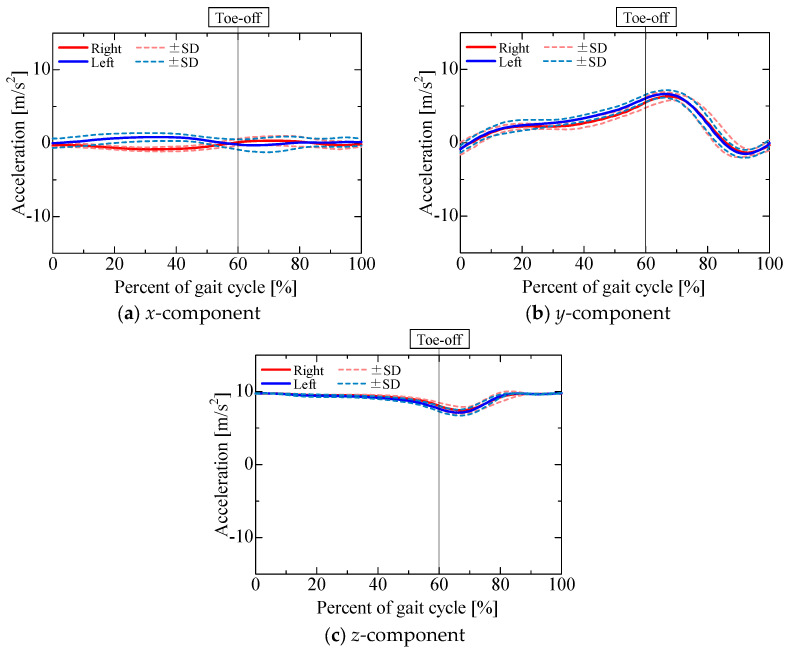
Gravitational acceleration.

**Figure 11:**
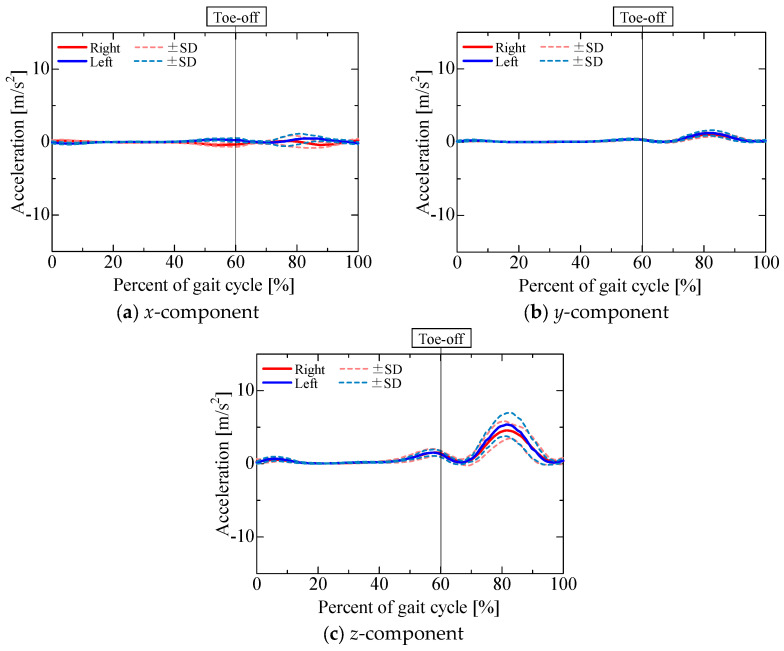
Centrifugal acceleration.

**Figure 12:**
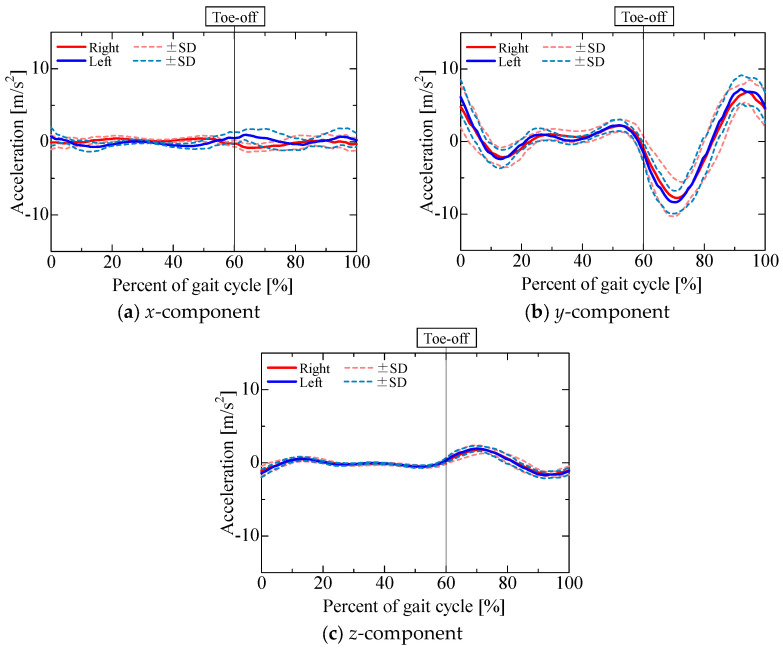
Tangential acceleration.

**Figure 13:**
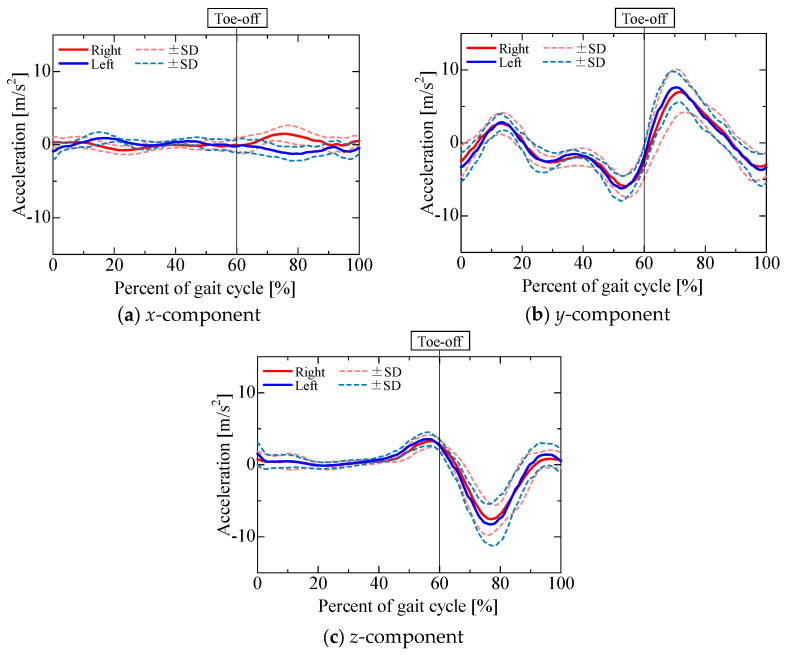
Translational acceleration.

**Figure 14:**
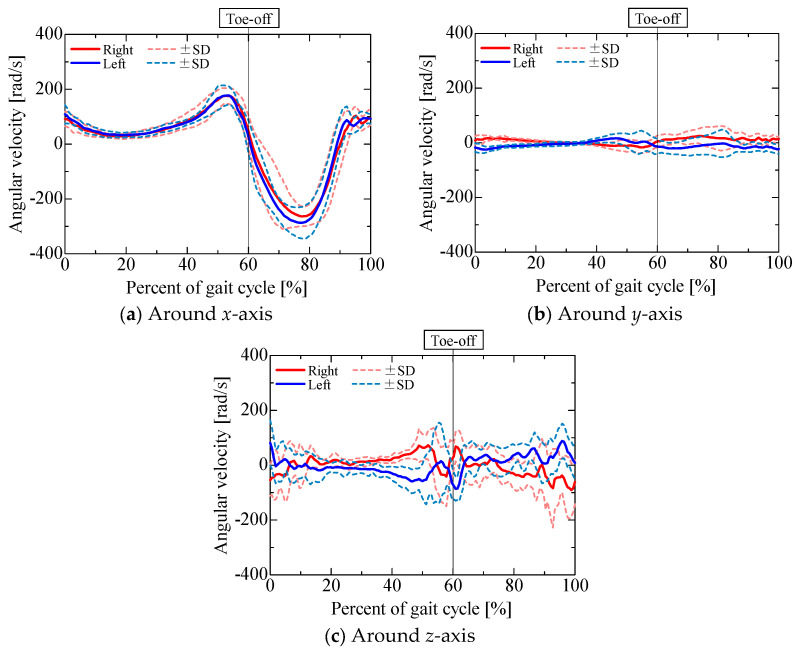
Gyroscope output obtained from nine-axis motion sensor.

The accelerometer output is shown in Figure 9. Figure 10, Figure 11, Figure 12 and Figure 13 show the gravitational, centrifugal, tangential, and translational acceleration gait cycle waveforms computed using the proposed method. In addition, to present the changes in centrifugal and tangential acceleration, the gyroscope output obtained from the nine-axis motion sensor is shown in Figure 14. The accelerations and gyroscope outputs were obtained during normal gait (3 trials for each of the 5 participants, for a total of 15 trials). The results are shown in the coordinate system of the nine-axis motion sensor attached to the lower leg. The *x*-axis is the lateral axis, with leftward being positive; the *y*-axis is the anteroposterior axis, with frontward being positive; and the *z*-axis is the longitudinal axis, with downward being positive. Thus, the orientation of the sensor coordinates changes relative to the walking direction as the posture of the lower leg changes.

The impact of pose estimation accuracy on subsequent acceleration decomposition manifests primarily in the translational and gravitational acceleration components derived from the pose estimation results. Assuming estimation errors of 1 degree in each of the roll, pitch, and yaw angles, the resulting errors in gravitational and translational accelerations are approximately 0.05 m/s^2^ along both the *x*- and *y*-axes and approximately 0.01 m/s^2^ along the *z*-axis. In contrast, centrifugal and tangential accelerations are computed solely from gyroscope data and therefore remain unaffected by errors in pose estimation.

## 4. Discussion

The output of the gyroscope attached to the lower leg, as shown in Figure 13, was found to be similar to that obtained in several previous studies [30,31,32], demonstrating that the angular velocity increases at the same time in the gait cycle regardless of walking speed when the walking speed is in the range of 0.4 to 1.6 m/s. In this study, the experimental conditions involved the participants walking using a natural stride in time with a metronome (90 bpm). Considering the stride length for each participant, the walking speed was approximately 1.05 to 1.2 m/s. It is thus reasonable that the angular velocity matched those reported in these previous studies. Therefore, the changes in centrifugal and tangential accelerations were examined by considering the gyroscope output and the knee rotational motion in each gait phase.

The *x*-component of the accelerometer output during the stance phase (Figure 9a) maintained a positive value for the left leg and a negative value for the right leg, similar to the *x*-component of the gravitational acceleration (Figure 10a). The *x*-component of the centrifugal acceleration remained close to zero throughout the stance phase. The correlation coefficients between the *x*-component of the accelerometer output and that of the gravitational acceleration during the stance phase were 0.50 ± 0.28 for the right leg and 0.53 ± 0.17 for the left leg. The root mean square errors (RMSEs) between the *x*-component of the accelerometer output and the corresponding component of the gravitational acceleration during the stance phase were 0.52 ± 0.15 m/s^2^ for the right leg and 0.52 ± 0.11 m/s^2^ for the left leg. For some participants, the *x*-component of the accelerometer output during the stance phase was mainly composed of the gravitational acceleration included in the posture change of the lower leg. However, not all participants presented this phenomenon. As shown in Figure 7b and Figure 8b, the lower leg tended to abduct slightly during the stance phase. The *x*-axis of the sensor for the right leg was oriented opposite to the direction of gravity, while that of the sensor for the left leg was oriented in the direction of gravity. Therefore, for some participants whose lower leg tended to abduct slightly during the stance phase, the *x*-axis component of the accelerometer output during the stance phase might include gravitational acceleration. In contrast, the *x*-component of the accelerometer output exhibited a negative peak for the left leg and a positive peak for the right leg in the middle swing phase, similar to the *x*-component of the translational acceleration (Figure 13a). However, the correlation coefficients between the *x*-component of the accelerometer output and that of the translational acceleration during the swing phase were 0.62 ± 0.37 for the right leg and 0.38 ± 0.40 for the left leg, while the root mean square errors (RMSEs) between the *x*-component of the accelerometer output and that of the translational acceleration during the swing phase were 0.97 ± 0.39 m/s^2^ for the right leg and 1.13 ± 0.37 m/s^2^ for the left leg. The large variability in the correlation coefficients among the participants suggests that the acceleration components contained in the accelerometer during the swing phase differ greatly between individuals.

The *y*-component of the accelerometer output (Figure 9b) changed slightly during the stance phase and increased in the positive direction in the pre-swing phase. As the changes in the *y*-component of the centrifugal acceleration were small, the *y*-component of the accelerometer output seemed to be mainly composed of the *y*-components of the gravitational (Figure 10b), tangential (Figure 12b), and translational (Figure 13b) accelerations. The *y*-axis of the sensor coordinate system became increasingly oriented to the direction of gravity with increasing knee flexion. The *y*-component of the accelerometer output increased as the y-component of gravitational acceleration reached its maximum at the initial swing phase. However, despite the *y*-component of gravitational acceleration decreasing with knee extension in the middle swing phase, the *y*-component of the accelerometer output remained positive until the terminal swing phase, which seemed to be due to the increase in the *y*-component of tangential acceleration associated with the angular acceleration as knee flexion increased, mainly toward the stance phase. The proportion of acceleration components included in the accelerometer output was found to change from moment to moment due to knee flexion and extension during the swing phase. Nevertheless, the correlation coefficients between the *y*-component of the accelerometer output and the sum of the *y*-components of the gravitational, tangential, and translational accelerations during the swing phase were 0.77 ± 0.11 for the right leg and 0.76 ± 0.13 for the left leg, while the associated root mean square errors (RMSEs) were 1.86 ± 0.55 m/s^2^ for the right leg and 1.67 ± 0.44 m/s^2^ for the left leg, confirming that the *y*-component of the accelerometer output is primarily composed of the *y*-components of gravitational, tangential, and translational accelerations.

The *z*-component of the accelerometer output (Figure 9c) remained near 10 m/s^2^. As the *z*-axis of the sensor coordinate system was oriented downward for most of the gait cycle, gravitational acceleration (Figure 10c) accounted for a large proportion of the overall acceleration. In contrast, the *z*-component of the accelerometer output increased in the negative direction from the initial to the middle swing phase, which seemed to be due to the influence of the *z*-component of gravitational acceleration (Figure 10c) as well as the *z*-component of translational acceleration (Figure 13c). The correlation coefficients between the *z*-component of the accelerometer output and the sum of the *z*-components of gravitational and translational accelerations from the initial to the middle swing phase were 0.94 ± 0.05 for the right leg and 0.96 ± 0.03 for the left leg, while the associated root mean square errors (RMSEs) were 1.33 ± 0.39 m/s^2^ for the right leg and 1.66 ± 0.47 m/s^2^ for the left leg. Therefore, the *z*-component of the accelerometer output from the initial to the middle swing phase is considered to be mainly composed of translational acceleration, caused by the rotational movement of the proximal joints, and gravitational acceleration.

The *x*-component of centrifugal acceleration hardly changed throughout the gait cycle (Figure 11a). The *x*-component of centrifugal acceleration is mainly due to rotational motion around the *y*- and *z*-axes in the sensor coordinates, which is caused by knee adduction and abduction, as well as internal–external rotation. From Figure 14, it can be seen that the angular velocities around the *y*- and *z*-axes were small compared to that around the *x*-axis, which was caused by knee flexion and extension. Thus, it is considered that the *x*-component of centrifugal acceleration hardly changed. The increases in the *y*- and *z*-components of centrifugal acceleration during the pre-swing and mid-swing phases are mainly due to knee flexion and extension (Figure 11b,c): as knee flexion and extension are rotational motions around the *x*-axis in the sensor coordinates, centrifugal acceleration occurs in the *y*- and *z*-axes in the sensor coordinates. The fact that the *y*-component of the centrifugal acceleration was smaller than the *z*-component was influenced by the position vector ***r*** from the sensor to the center of the knee joint. In this experiment, $r_{x}$ = 0 m, $r_{y}$ = −0.05 m, and $r_{z}$ = −0.215 ~ −0.190 m. To calculate the *z*-component, the square of the angular velocity *ω_x_* around the *x*-axis was multiplied by $r_{z}$, while, to calculate the *y*-component, the square of *ω_x_* was multiplied by $r_{y}$. As the magnitude of the acceleration is affected by the distance from the sensor to the center of the knee joint in each axis, the *y*-component of the centrifugal acceleration was smaller than the *z*-component. However, as the *y*- and *z*-components of centrifugal acceleration were caused by knee flexion and extension, both components seemed to be important accelerations relating to the rotational motion of the knee during walking. When we focused on the magnitude of acceleration throughout the gait cycle, we found that the peak values for both the *y*- and *z*-components in the mid-swing phase were larger than those in the pre-swing phase. The result indicates that the angular velocity for knee extension in the mid-swing phase is larger than the angular velocity for knee flexion in the pre-swing phase. Therefore, the gait phase in which peaks occur in the *y*- and *z*-components of centrifugal acceleration, as well as the difference between the magnitudes of the peak values, might be useful as indicators demonstrating the characteristics of normal gait.

The *x*-component of the tangential acceleration hardly changed throughout the gait cycle (Figure 12a). The *x*-component of tangential acceleration, as for the *x*-component of centrifugal acceleration, was mainly due to rotational motion around the *y*- and *z*-axes in the sensor coordinates, which is caused by knee adduction and abduction as well as internal–external rotation. Figure 14 shows that the angular velocities around the *y*- and *z*-axes were small compared to that around the *x*-axis, which was due to knee flexion and extension. Thus, it is considered that the *x*-component of tangential acceleration hardly changed. The increase in the negative direction of the *y*-component and the increase in the positive direction of the *z*-component in the initial swing phase were mainly due to knee flexion and extension (Figure 12b,c). As knee flexion and extension are rotational motions around the *x*-axis in the sensor coordinates, tangential acceleration occurs in the *y*- and *z*-axes in the sensor coordinates. The fact that the *z*-component of the tangential acceleration was smaller than the *y*-component indicates the influence of the position vector ***r*** from the sensor to the center of the knee joint, as observed for centrifugal acceleration. To calculate the *y*-component, the angular acceleration $\dot{\omega_{x}}$ was multiplied by $r_{z}$, while, to calculate the *z*-component, $\dot{\omega_{x}}$ was multiplied by $r_{y}$. As the magnitude of the acceleration is affected by the distance from the sensor to the center of the knee joint in each axis, the *z*-component was smaller than the *y*-component. However, as the *y*- and *z*-components of tangential acceleration were caused by knee flexion and extension, both components seemed to be important accelerations relating to the rotational motion of the knee during walking. The increase in the negative direction of the *y*-component and the increase in the positive direction of the *z*-component in the initial swing phase were caused by angular accelerations during knee extension; these exhibited peaks earlier than the *y*- and *z*-components of the centrifugal acceleration, which were calculated using the angular velocity.

As described above, the components of the output of the accelerometer attached to the lower leg were confirmed. As the *z*-axis of the sensor coordinate system, aligned with the longitudinal axis of the lower leg, was pointing vertically downward for most of the gait cycle, it is obvious that the proportion of gravitational acceleration in the *z*-component of the accelerometer output was large. However, as the gravitational acceleration contained in the accelerometer output changes from moment to moment with changes in the posture of the lower leg, in order to use the acceleration generated during walking for gait evaluation, it is necessary to determine the components of the accelerometer output as time-series data. Although it is well established that the lower leg’s acceleration varies significantly during the swing phase, the results of this study demonstrated that, in the anterior direction of the lower leg, the acceleration output during the first half of the swing phase is primarily influenced by gravitational acceleration, tangential acceleration, and translational acceleration. In the longitudinal direction, the acceleration output is predominantly determined by gravitational acceleration throughout the gait cycle, and additionally by translational acceleration during the first half of the swing phase.

In this study, we analyzed the components of acceleration present in the output of accelerometers mounted on the anterior aspect of the lower leg during each phase of the gait cycle. Variations in gravitational acceleration were attributed to changes in the posture of the segment to which the sensor was attached. Centrifugal and tangential accelerations resulted from movements of the proximal joints adjacent to the sensor location, whereas translational acceleration was generated by motions of joints situated further proximally. By decomposing the accelerometer output into these distinct components, it becomes possible to capture characteristics of limb motion that cannot be detected solely through changes in posture or joint angles. Based on these insights, the findings of this study have potential for use in applications such as evaluating rehabilitation outcomes and identifying gait abnormalities that may not be discernible through visual observation.

## 5. Conclusions

This study examined the normal gait of participants according to the gravitational, centrifugal, tangential, and translational accelerations of their lower legs. As a result, we reached the following conclusions:

1. The acceleration components contained in the output of accelerometers attached to the lower legs were described. The gravitational acceleration contained in the accelerometer output was determined by successively estimating the sensor pose. The translational acceleration was indicated in terms of the centrifugal and tangential accelerations, which were both calculated from the gyroscope output and the gravitational acceleration. The characteristics of normal gait were assessed according to the acceleration components.
2. These results demonstrated that the lateral acceleration output during the swing phase is dominated by translational acceleration, which implies that it might be due to acceleration caused by the left–right shift of the center of gravity. The anteroposterior direction acceleration output was dominated by gravitational acceleration in the first half of the swing phase and tangential acceleration in the second half, which implies that it might be due to the change in lower-leg posture in the first half of the swing phase and knee extension in the second half of the swing phase. The acceleration output in the longitudinal direction of the lower leg was dominated by gravitational acceleration throughout the gait cycle and by translational acceleration in the first half of the swing phase, which implies that it might be affected by the acceleration caused by the up–down shift of the center of gravity in the first half of the swing phase.

The output of an accelerometer attached to the lower leg was decomposed into its individual components, confirming the feasibility of using acceleration data generated only from walking motion for gait evaluation. Several avenues for extension of this approach can be considered. Although the ultimate objective was to evaluate gait using only motion-induced acceleration, this study was limited to healthy adults walking at a fixed cadence. However, as the proposed method utilizes 3D pose estimation to perform downstream acceleration decomposition, it appears to be adaptable to abnormal gait patterns, including those involving large movements in the frontal and transverse planes. Another potential direction involves extending the analysis to more proximal joints or integrating the method with complementary sensing modalities. In the field of industrial robotics, adapting this framework to exoskeleton control or joint torque estimation could enhance its practical impact. Nonetheless, before applying the method to kinetic analysis, its validity must first be confirmed for body segments that are more proximal than the lower leg.

## Figures and Tables

**Table 1:** Root mean square errors for results obtained from nine-axis motion sensor and 3D motion analysis system [°].

Sensor	Roll	Pitch	Yaw
Sensor1 (Right)	5.15 ± 0.96	1.88 ± 0.43	5.40 ± 1.79
Sensor2 (Left)	4.28 ± 0.61	2.44 ± 0.63	5.79 ± 0.93

## Data Availability

The raw data supporting the conclusions of this article will be made available by the authors on request.
